# Radiostereometry and new prostheses

**DOI:** 10.3109/17453674.2012.678796

**Published:** 2012-04-24

**Authors:** Edward Valstar, Bart Kaptein, Rob Nelissen

**Affiliations:** Organisers, Second International RSA Meeting

The Second International RSA Meeting was held in Leiden, the Netherlands, in April 2011. The meeting was attended by 110 participants from 12 countries, including the USA, Canada, and not least Sweden—the country in which the clinical use of RSA was initiated by Göran Selvik. The large worldwide attendance of the meeting clearly indicates that the use of RSA is spreading across the world. It has not only attracted the attention of surgeons, researchers, and the orthopedics industry but also that of the regulatory bodies in Europe, the United States, and Australia. There is growing awareness that new joint replacement prostheses, cements, and surgical techniques should be thoroughly evaluated before general release onto the market. Thus, the rather erratic introduction of new prostheses or new procedures should be replaced by a phased introduction, an approach that has been mandatory in the pharmaceutical industry for decades (Nelissen et al. 2011).

One would expect that disasters such as with Boneloc cement and the Capital hip prosthesis could not happen today. However, in general the introduction of new prostheses is still done in almost the same way as it was 20 years ago. More than 25 years ago, Malchau (1995) proposed a controlled, stepwise introduction of new prostheses. This message has been given many times, even in editorials in Acta (Bauer 1992, Kärrholm 2003). However, recent problems with the Proxilock hip, the ASR hip, the Accord knee, and the St. Leger knee illustrate the urgent need for improved early evaluation of new prostheses—a process best performed in cooperation between the orthopedic community, industry, and regulators.

The potential of RSA as a method of early (pre-introductory) assessment of implant performance is substantial. This potential is currently being recognized by various regulatory bodies at different levels. The NICE guidelines of 2003 (UK) require adequate long-term clinical data for hip prostheses and suggest RSA as a promising technique that may be an alternative for long-term follow-up studies. However, additional proof of its predictive value regarding future loosening is not only advised but also demanded by regulatory bodies. In its new guidelines for hip prostheses, the Dutch Orthopaedic Society has stated that any new hip prosthesis that is being considered for (commercial) introduction to the Dutch market must go through a phased introduction (Swierstra et al. 2011). This phased introduction includes mandatory RSA studies even before larger clinical trials can be started.

Mandatory RSA studies require that the results of different RSA studies can be compared. Thus, an international RSA group published the first RSA standardization paper in Acta Orthopaedica (Valstar et al. 2005), and a larger consortium with RSA experts from all over the world is now establishing an actual ISO standard for RSA. For ISO insiders: the draft of the standard is now labeled Draft International Standard and is currently being reviewed by all member states. The standard is expected to be finalized this year. In addition, an international RSA network is currently being established (www.rsaresearch.org). This network is intended to be a platform for improvement of the quality of clinical radiostereometry research by sharing knowledge between research groups.

4 papers that were presented at the Second International RSA meeting, and selected by an international jury of RSA experts to be of particular interest, have been accepted for publication in Acta Orthopaedica. 1 of them (Wilson et al. 2012) was published in the February issue, and the other 3 are published in this April issue (Aro et al. 2012, Nebergall et al. 2012, Nieuwenhuijse et al. 2012). Furthermore, 5 other RSA studies are published in the current issue (Bohm et al. 2012, Pijls et al 2012a, b, Röhrl et al. 2012, von Schewelov et al. 2012).

3 of the papers in this issue of Acta present medium- to long-term RSA results of hip and knee prostheses. It is good to see that the number of medium- to long-term RSA studies is increasing, as this will lead to a better understanding of the predictive value of short-term RSA results for future implant failure by aseptic loosening. Nieuwenhuijse et al. (2012) present a 10–12 year follow-up study of the Exeter stem. Pijls et al. (2012a) present a study on the effect of HA coating on the long-term fixation (up to 16 years) of the Interax total knee prosthesis, and Röhrl et al. (2012) present the 10-year results of submelt-annealed highly crosslinked polyethylene cemented cups.

2 papers focus on the relationship between bone mineral density and stem migration. The study by Aro et al. (2012) established the relationship between low BMD and increased stem migration in cementless total hip arthroplasty in a cohort of 57 female patients, and the study by von Schewelov et al. (2012) concerns the fixation of a fully HA-coated Corail stem for treatment of dislocated femoral neck fractures.

2 other papers deal with the effect on prosthetic migration of adding tobramycin to a bone cement (Bohm et al. 2012), and a meta-analysis confirms the predictive value of RSA for the high failure rate of the uncoated Interax prosthesis (Pijls et al. 2012b.).

The paper by Nebergall et al. (2012) describes a new application of RSA, which was used to evaluate a transdermal, femoral implant system in above-the-knee amputee patients in whom the use of a conventional socket prosthesis was difficult if not impossible.

It is good to see that RSA is spreading across the world and that the number of long-term follow-up studies is increasing. Such further studies may support the value of RSA for early prediction of prosthetic loosening; compulsory early RSA studies will then be part of a phased introduction of new implants.

The large number of research groups that are involved in RSA and the current developments call for another RSA meeting. The next biannual International RSA Meeting is scheduled to take place in 2013, and will celebrate 40 years of RSA. We are very happy that this meeting will take place in Lund, Sweden, where it all started.
